# Dr. H. R. Robards

**Published:** 1861-01

**Authors:** 


DR. H. R. ROBARDS, late Professor of Surgery in the Memphis Medical College, died on the eighteenth of October.

